# Incorporating patient-reported outcome measures (PROMs) into a clinical quality registry (CQR) for ovarian cancer: considerations and challenges

**DOI:** 10.1186/s12913-024-11042-8

**Published:** 2024-07-08

**Authors:** Yael R Lefkovits, Natalie Heriot, Alice Sporik, Sharnel Perera, Michael Friedlander, Cyril Dixon, Paul A Cohen, Yeh Chen Lee, Simon Hyde, Gary Richardson, Penelope Webb, Robert Rome, Madeleine King, John Zalcberg, Penelope Schofield

**Affiliations:** 1 Royal Melbourne Hospital, Parkville, Melbourne, Victoria Australia; 2 School of Public Health and Preventive Medicine, Faculty of Medicine, Monash University, Melbourne, Australia; 3 Sir Peter MacCallum Department of Oncology, The University of Melbourne, Parkville, Melbourne, Victoria Australia; 4 Department of Medical Oncology, University of New South Wales, Sydney, Australia; 5 Ovarian Cancer Australia, Melbourne, Victoria Australia; 6 Discipline of Obstetrics and Gynaecology, Medical School, University of Western Australia, Crawley, Western Australia Australia; 7 Department of Gynaecological Oncology, St John of God Subiaco Hospital, Subiaco, Western Australia Australia; 8 Institute of Health Research, University of Notre Dame, Fremantle, Western Australia Australia; 9 Medical Oncology Department, Prince of Wales Hospital, Randwick, New South Wales Australia; 10 Faculty of Medicine and Health, University of New South Wales, Randwick, New South Wales Australia; 11 NHMRC Clinical Trials Centre, University of Sydney, Camperdown, New South Wales Australia; 12 Department of Gynaecological Oncology, Mercy Hospital for Women, Heidelberg, Victoria Australia; 13 Szalmuk Family Department of Medical Oncology, Cabrini Research, Malvern, Victoria Australia; 14 QIMR Berghofer Medical Research Institute, Herston, Queensland Australia; 15 Clinical Institute of Obstetrics and Gynaecology, Epworth HealthCare, East Melbourne, Victoria Australia; 16 School of Psychology, University of Sydney, Sydney, NSW Australia; 17 Behavioural Sciences Unit, Health Services Research and Implementation Sciences, Peter MacCallum Centre, Melbourne, Victoria Australia; 18 Department of Psychology and Iverson Health Innovation Research Institute, Swinburne University, Melbourne, Victoria Australia; 19 Department of Behavioural Sciences in Cancer, Swinburne University, Hawthorne, Victoria Australia

**Keywords:** Patient reported outcome measures, Gynaecological cancer, Clinical quality registry, Ovarian cancer, Quality of life

## Abstract

As medical treatment increasingly focuses on improving health-related quality of life, patient-reported outcome measures (PROMs) are an essential component of clinical research. The National Gynae-Oncology Registry (NGOR) is an Australian clinical quality registry. A suitable PROM was required for the NGOR ovarian cancer module to complement clinical outcomes and provide insights into outcomes important to patients. Our narrative review aimed to identify existing ovarian cancer-specific PROMs and ascertain which tool would be most appropriate for implementation into the NGOR ovarian cancer module.

A literature review of Cochrane Library, Embase, MEDLINE and PubMed databases was performed to identify existing ovarian cancer-specific PROM tools. A steering committee was convened to (1) determine the purpose of, and criteria for our required PROM; and (2) to review the available tools against the criteria and recommend the most appropriate one for implementation within the NGOR.

The literature review yielded five tools: MOST, EORTC QLQ-OV28, FACIT-O, NFOSI-18 and QOL-OVCA. All were developed and validated for use in clinical trials, but none had been validated for use in clinical quality registry. Our expert steering committee pre-determined purpose of a PROM tool for use within the NGOR was to enable cross-service comparison and benchmarking to drive quality improvements. They identified that while there was no ideal, pre-existing, ovarian cancer-specific PROM tool for implementation into the NGOR, on the basis of its psychometric properties, its available translations, its length and its ability to be adapted, the EORTC tool is most fit-for-purpose for integration into the NGOR.

This process enabled identification of the tool most appropriate to provide insights into how ovarian cancer treatments impact patients’ quality of life and permit benchmarking across health services.

## Background

Historically, the goal of oncology treatment has been to increase the duration of life, with ‘survival time’ as a traditional outcome of interest in clinical trials [1]. In the past few decades, health-related quality of life (HRQL) has become a key priority, no less so than for patients with ovarian cancer given the significant morbidity associated with the disease and its treatment. HRQL is a multidimensional construct with domains related to mental, physical, emotional and social functioning and provides insight into the patient experience of illness including the effects of treatment [2]. Measuring it, in addition to traditional clinical outcomes, represents a paradigmatic shift in what constitutes ‘success’ in medicine [2]. As research trends progress from only including ‘hard’ endpoints, such as morbidity and mortality, HRQL data adds a critical new dimension of patients’ experience of illness, capturing outcomes that are fundamentally important from their perspective [3].

Unlike traditional clinical outcome indicators such as overall survival and progression-free survival, patient-reported outcome measures (PROMs) provide a valid and reliable assessment of symptoms and core domains of HRQL including physical, emotional and social functioning [4]. While historically PROMs have been used in research, they can also be used in the clinic to improve patient-provider communication, improve the monitoring of treatment responses and significantly improve patient satisfaction by identifying the outcomes that matter to patients [5]. Furthermore, PROMs have the potential to inform policy development aimed at reducing the economic and physical burden associated with poor HRQL, with such data possibly being useful for cost-benefit and cost-utility studies [6, 7].

Clinical Quality Registries (CQRs) are longitudinal databases which collect information on the quality of health-care within specific clinical domains by routinely amassing, analysing and reporting health-related information on quality indicators which reflect agreed best practice [8]. The purpose of these registries extends beyond the quality of care; they also identify directions for health services design and research. With increasing treatments and complex treatment protocols, there is a lag in health service delivery to respond to these rapid innovations. Registries are an opportunity to identify these impacts on a large scale.

In Australia, the Australian Commission of Safety and Quality in Health Care have developed an infrastructure model for best-practice development, design and operation of Australian CQRs [9]. They are increasingly utilised to benchmark clinical practice and outcomes of particular diseases across various centres and regions [8]. These comparisons can provide valuable indications of the standard of healthcare delivery, the safety and efficacy of treatment and whether or not the care is delivered in line with best practice guidelines [8].

‘Best practice’ in the medical sphere is generally understood to represent a standard of care considered optimal based on the existing evidence [10]. For ovarian cancer, a number of peer-reviewed, evidence-based international and Australian guidelines provide ‘best practice’ around diagnosis and treatment [11, 12]. Although some bodies are yet to include PROMs as a necessary component of their guidelines, other organisations such as the Food and Drug Administration (FDA), the European Society for Medical Oncology (ESMO) and the Consolidated Standard of Reported Clinical Trials (CONSORT) have all recommended the use of PROMs to augment clinical-level data from clinical trials [13–15].

To measure the performance of individual hospitals and health services in the real-world, CQRs assess the degree to which each hospital or health service adheres to best practice in the diagnosis, staging and management of patients with ovarian cancer. Traditionally, these registries measure the relationship between compliance with a series of structural or process quality indicators against customary clinical endpoints, such as disease recurrence and survival. Recommendations to incorporate PROMs into core-outcome sets in cancer care, as is recommended by the International Consortium for Health Outcome Measurement (ICHOM) initiative, reflects the notion that PROM data can be used to improve the quality of patient care [16]. Consequently, a number of CQRs in Australia are increasingly using PROMs to measure the HRQL of participants.

The National Gynae-Oncology Registry (NGOR) was established in 2017 and is a multi-module CQR that monitors the quality of care provided to women with gynaecological cancers across several states in Australia [17]. The first of four modules assesses the quality of care provided to, and the outcomes of women with primary epithelial ovarian, tubal and peritoneal cancers. The remaining modules covering the other key tumour sites (endometrial cancer, cervical cancer and cancers of the vulvar) are currently in development. The NGOR is planning to incorporate a suitable PROM tool into the registry’s routine data collection to complement the clinical data. The identification of poorer HRQL outcomes determined through benchmarking aggregate PROMs data against other similar health services, may enable evaluation of clinical management paradigms at that health service. As a simple example, if patients treated at a particular health service experienced on average, comparatively higher levels of post-surgical pain compared to their peers, clinicians at that service could implement improved pain management protocols after surgery.

To date, several PROM tools have been validated for use in epithelial ovarian cancer. However, these tools have been primarily intended and validated for use in clinical trials rather than in CQRs. This narrative review aimed to identify the existing PROM tools suitable for incorporation into the NGOR and evaluate the existing evidence supporting their validity and reliability for use within a CQR, with tool selection being guided by an expert steering committee.

## Main text

To address the aim, three complementary approaches were undertaken: (1) a comprehensive literature review; (2) assessment the psychometric properties of the various PROM tools; and (3) an expert steering committee review of the available ovarian cancer-specific PROM tools and identification of which tool is most adaptable and fit-for-purpose for incorporation into the NGOR.

### Literature review

To identify studies for inclusion in this review, three electronic databases (MEDLINE Complete, Embase and Cochrane Library) were searched from inception to the date of the search (between 1st November 2019 and 31st August 2020). Keywords used in the search were developed by the authors and included *ovarian neoplasm*, *ovarian cancer, patient reported outcome measures* and *quality of life*. The reference lists of papers meeting inclusion criteria were scanned for additional studies for potential inclusion in this review. No limitations were placed on the type of study included or the publication date. Review articles, pilot and feasibility studies were eligible for inclusion.

**Fig. 1:**
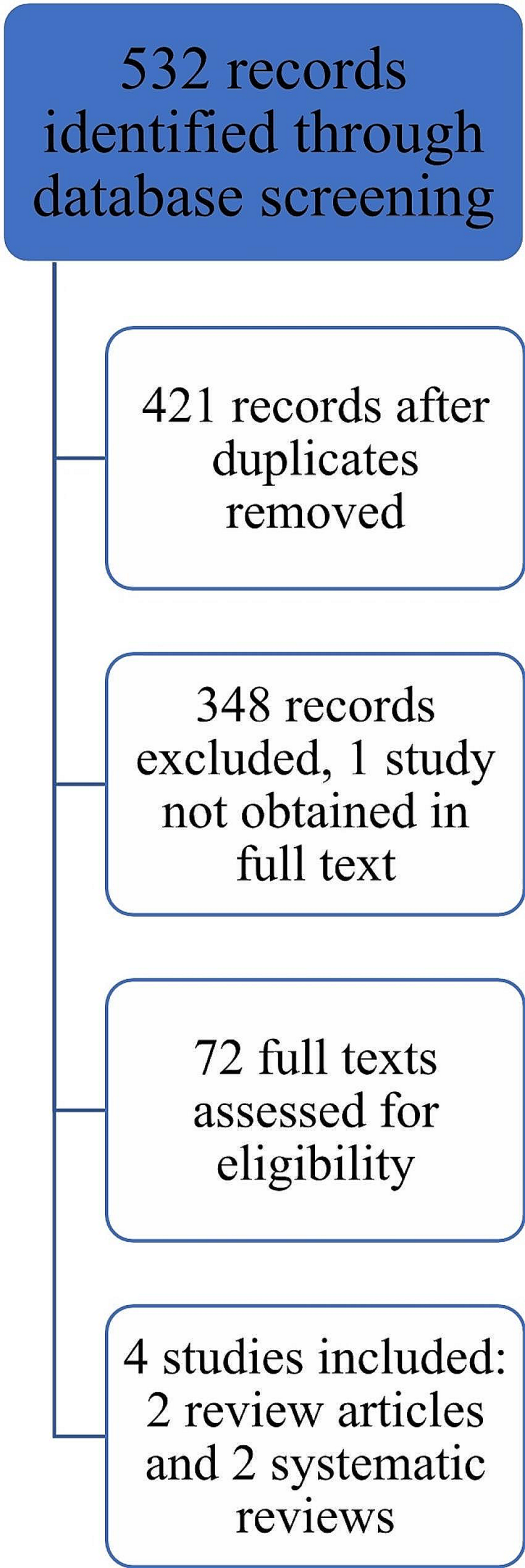
Flow diagram of the literature review process for studies comparing ovarian cancer-specific patient reported outcome measure tools

Studies were included in the literature review if they met the following criteria: (i) they focused on PROM tools relating to ovarian cancer or additional cancer types where results for an ovarian cancer subgroup were reported separately, (ii) they were published in English. Publications were imported into a database and all abstracts were assessed for relevance. The full texts of the papers classified as relevant were reviewed by two authors to determine their final classification as relevant or not relevant. Data were abstracted from relevant articles into an Excel spreadsheet including a list of all ovarian cancer-specific PROM tools. These tools were examined according to reliability, content-validity, consumer feedback and scope of metrics examined. PROM tools were only included if data on these metrics were available. Further, studies which included direct comparison of the ovarian-cancer specific tools and their metrics were included. A flowsheet outlining this process is included in Fig. 1.

### Assessment of PROM psychometric properties

After obtaining full text copies and examining 72 articles, we identified five ovarian cancer-specific PROM tools that have been validated for use in clinical trials. Only four studies were identified which provided direct comparison between existing ovarian-cancer specific PROM tools. These comparisons, as well as evidence regarding their reliability, validity, interpretability and responsiveness of the PROM tools, can be found in Table 1. These four psychometric criteria (reliability, validity, interpretability and responsiveness) are based mainly on the Consensus-Based Standards for the selection of health Measurement Instruments (COSMIN) guidelines [18] as well as the International Society for Quality of Life Research (ISOQOL) guidelines [19] which aim to improve the selection of outcome measurement instruments by developing tools for selecting the most suitable instrument for a particular situation.

**Table 1:** Studies comparing the reliability, validity and evidence supporting the use of ovarian cancer-specific PROM tools within a clinical trial setting

Study	PROM Specific Tool	Study Type	Conclusions
Preston et al., 2015 [32]	EORTC QLQ- OV28 FACT-O NFOSI-18	Systematic Review	Both the EORTC QLQ-OV28 and FACT-O had been extensively psychometrically tested, with a greater degree of evidence favouring the content validity and responsiveness of the FACT-0. However, given that the EORTC QLQ-OV28 has been tested in a greater number of studies, with almost twice the number of study participants, it is considered this tool to be the most robust ovarian cancer-specific PROM tool.
Luckett et al., 2010 [30]	EORTC QLQ-OV28 FACT-O NFOSI-18	Systematic Review	This study reported a superior quantity and quality of data supporting the use of FACT-O when compared to EORTC QLQ-OV28. Yet overall, Luckett el al found little evidence to favour the use of the EORTC questionnaires over the FACIT questionnaires or vice versa.
Jensen et al., 2013 [31]	EORTC QLQ-OV28 FACT-O NFOSI-18 QLQ-OVCA	Review Article	This study stated that although all four tools have been demonstrated to be valid and reliable ovarian cancer-specific HRQL measures, the only tool that had direct input from patients in its development was NFOSI-18. However, it is worth noting that is also less psychometrically tested than its original counterpart, FACT-O.
King et al., 2014 [25]	EORTC QLQ-OV28 FACT-O NFOSI-18	Review Article	The study aimed to determine the optimal PROM measure for use in trials of palliative chemotherapy for women with symptomatic ovarian cancer. It concluded that none of the four existing tools met all of their defined optimality criteria (content validity, recall period, numeral rating scale for items and symptom index scoring). As such, they proposed the development of a new tool, MOST, for use in trials of palliative chemotherapy for women with symptomatic ovarian cancer.

The COSMIN guideline’s, Risk of Bias Checklist includes ten domains by which to measure the suitability of a PROM tool including PROM development, content validity, structural validity, internal consistency, cross-cultural validity, reliability, measurement error, criterion validity, hypothesis testing for construct validity and responsiveness [18]. Each item is given a rating from very good to inadequate (gradings including very good, adequate, doubtful, inadequate and not applicable). The ISOQOL stipulates that to meet the minimum standard for a PROM tool, there needs to be documentation of the conceptual and measurement model, evidence of reliability, validity (content and construct), interpretability of scores and quality translations available and there is an acceptable patient and investigator burden [19].

We did not perform our own psychometric analyses of the individual PROM tools, but rather reviewed the existing studies which have assessed the validity, reliability, functionality/responsiveness and interpretability of the existing tools [Table 2]. Therefore, many of the domains in the ISOQOL and COSMIN guidelines were not able to be assessed as they were not referenced or included in the existing studies assessing the ovarian-cancer specific PROM tools. Each tool was assessed for the four aforementioned domains in addition to the presence of translations and their patient and investigator burden. However, measurement error, cross-cultural validity and internal consistency were not routinely reviewed.

**Table 2:** Psychometric properties of each of the five ovarian cancer-specific PROM tools

PROM tool	Interpretability and functionality	Reliability	Validity
EORTC QLQ-OV28	Covers many of the most common and relevant symptoms for women with ovarian cancer. During the developmental stage, direct consumer input was used to formulate the symptoms and concerns deemed to be most important to women with ovarian cancer *COSMIN rating: Very good*	Strong evidence supporting its reliability in a clinical trial setting including satisfactory internal consistency and test-retest reliability as per Preston et al. and Luckett et al’s systematic reviews *COSMIN rating: Very good*	Meets all the necessary symptoms for content validity as per King et al’s symptom set for content validity for a PROM tool for women with ovarian cancer. However, these symptoms are split into numerous scales, dissipating the potential symptom benefit signal. This tool has some evidence regarding construct, content and criterion validity as per Preston et al’s systematic review *COSMIN rating: Adequate*
NFOSI-18	Favoured for its brevity and the focused symptom measurement for advanced ovarian cancer. It contains a number of important questions around sexuality, concerns over reproductive function and ability to work. During the developmental stage, direct consumer input was used to formulate the symptoms and concerns deemed to be most important to women with ovarian cancer *COSMIN rating: Very good*	Strong evidence supporting its reliability in a clinical trial setting as per Preston et al. and Luckett et al’s systematic reviews *COSMIN rating: Very good*	Meets most but not all of the necessary symptoms for content validity with a score of 6/10 as per King et al’s symptom set for content validity for a PROM tool for women with ovarian cancer. However, it has been demonstrated to have content validity in other studies including Luckett et al’s systematic review. This tool also has good evidence regarding construct, content and criterion validity as per Preston et al’s systematic review *COSMIN rating: Adequate*
FACT-O	Includes important metrics such as measurement of pain, fatigue and overall QOL. Some women preferred FACT-O for its ease of use, ability to be completed in five minutes without assistance and the fact that they can weight the category of questions deemed most important to their lives. Other women found questions about sexuality to be ‘intrusive.’ Consumers did not have direct input in selecting the items for inclusion in this tool *COSMIN rating: Doubtful*	Strong evidence supporting its reliability in a clinical trial setting including satisfactory internal consistency and test-retest reliability as per Preston et al. and Luckett et al’s systematic reviews *COSMIN rating: Very good*	Meets most, but not all of the necessary symptoms for content validity with a score of 8/10 as per King et al’s symptom set for content validity for a PROM tool for women with ovarian cancer. However, it has been demonstrated to have content validity in other studies including Luckett et al’s systematic review. This tool also has good evidence regarding construct, content and criterion validity as per Preston et al’s systematic review *COSMIN rating: Adequate*
QOL-OVCA	Contains a number of items felt to be poorly understood by respondents including items such as survivorship guilt. Consumers did not have direct input in selecting the items for inclusion in this tool *COSMIN rating: Inadequate*	Some evidence supporting its reliability in a clinical trial setting *COSMIN rating: Adequate*	Not discussed in King et al’s review on content validity. However, it has been demonstrated to have content validity in other studies including Luckett et al’s systematic review *COSMIN rating: Adequate*
MOST	During the developmental stage, direct consumer input was used to formulate the symptoms and concerns deemed to be most important to women with ovarian cancer *COSMIN rating: Very good*	Some evidence supporting its reliability in a clinical trial setting, specifically, palliative chemotherapy in clinical trials for ovarian cancer *COSMIN rating: Adequate*	Meets all the necessary symptoms for content validity as per King et al’s symptom set for content validity for a PROM tool for women with ovarian cancer *COSMIN rating: Adequate*

The systematic reviews discussed in this table include Luckett et al’s systematic review [29] which assessed the various ovarian cancer-specific PROM tools and their psychometric profiles as well as Preston et al’s systematic review [31] which similarly assessed three ovarian cancer specific PROM tools (EORTC QLQ-OV28, NFSOI-18 AND FACT-O) and analysed their psychometric qualities. The data on interpretability, functionality and reliability is based off these aforementioned systematic reviews. Validity: King et al. [24] applied qualitative and quantitative methods to data from stage 1 of the Gynacologic Cancer Intergroup Symptom Benefit Study to determine the set of necessary symptoms to objectively assess candidate PROMs against the optimality criteria. Ten symptoms were identified including pain, fatigue, abdominal bloating/discomfort, sleep disturbance, bowel disturbance, nausea and vomiting, shortness of breath, poor appetite, urinary symptoms and weight changes

### Expert steering committee

Although the reliability, content-validity and interpretability of each tool are important to ascertain, they are not the only metrics upon which a decision of which tool to incorporate into a CQR, can be made. Given incorporation of a PROM tool within an ovarian cancer-specific registry is a relatively novel concept, a multi-disciplinary steering committee and advisory body was created to identify which of these PROM tools was best suited for incorporation into the NGOR and adaptable for use within a CQR.

The committee, comprised of twenty members, included three patients with ovarian cancer (consumers), epidemiologists, medical oncologists, junior doctors, gynaecological oncologists, PROMs methodologists, behavioural scientists, and representatives from Ovarian Cancer Australia (OCA), the leading national consumer organisation that advocates for women affected by ovarian cancer. Committee members were selected on the basis of their expertise and interest with the steering committee aiming to include the perspective of women with ovarian cancer, their treating physicians (both medical and surgical), researchers with expertise in PROM tools, epidemiologists with expertise in CQRs and members of the OCA executive team with expertise in the care needs and provision of multi-disciplinary treatment to women with ovarian cancer. All the selected epidemiologists had extensive experience with either the development and implementation of CQRs or with PROM tools and oncologists selected to the committee had special interests or experience with ovarian cancer. Consumer representatives all had prior experience with ovarian cancer advocacy in clinical research settings.

The purpose of the expert steering committee was to identify which of the candidate tools would best allow for cross-service comparison (benchmarking) to assess variation among health services to drive quality improvement and which of the existing ovarian cancer-specific PROM tools would be most suitability for implementation into the NGOR. Over a nine-month period, the committee met on a monthly basis to discuss the aims of a PROM tool within the NGOR, identify key patient-reported outcomes of interest, review the available ovarian-cancer specific PROM tools and identify if any of the tools are logistically feasible and fit-for-purpose for integration into the NGOR. In addition to the committee identifying a set of PROMs which they believed would best improve the quality of care for patients with ovarian cancer within and between various organisations, the committee also aimed to analyse how best to use the data from these PROMs, so they are actionable by clinicians and institutions.

### Ovarian cancer-specific PROM tools

Our review of the literature yielded five ovarian-cancer specific PROM tools, each reviewed in detail in the following section. Our researchers then aimed to identify published studies examining the psychometric properties of these existing PROM tools and provide a comparison between the tools. As outlined in Fig. 1, we identified 72 full texts for reviews and four studies ultimately met our inclusion criteria: two systematic reviews and two review articles. In addition to considering the psychometric data analyses of each of the ovarian cancer-specific PROM tools, our committee evaluated a number of other domains including the length of the tools, the content of the tools (i.e., which symptom domains they covered) [Table 3], the number and quality of translations and the adaptability for use within a CQR to identify which of the available PROM tools is most fit-for-purpose for use within the NGOR.

**Table 3:** Symptoms and concerns assessed in each ovarian cancer-specific PROM tool *–* number of items per tool

Symptoms / concerns	EORTC QLQ-C30/QLQ-OV28	FACT-0	NFOSI-18	QLQ-OVCA	MOSTv2
Gastrointestinal	12	6	6	3	8
Gentio-urinary	1				1
Systemic Symptoms / energy	5	4	4	2	3
Neuropathy	2			1	1
Dermatological complaints	1		1		1
Reproductive issues	2	1		2	
Body image		2		1	
Hair loss	2	1	1		1
Emotional well-being	4	10	3	20	3
Spirituality/religion				3	
Sex drive / sexuality		2		1	
Impact on finances	1	1		2	
Impact on relationships	2	7		4	
Impact on sleep	1	1	1	1	1
Survivorship Guilt				1	
Impact on functioning / independence	9	3	1	2	3
Pain	3	1	1	1	2
Cognition	2			1	

Gastrointestinal symptoms were defined as any symptom, sign or concern relating to the gastrointestinal, oesophageal or hepatobiliary system. Systemic symptoms were defined as signs, symptoms or concerns affecting a number of organs or tissues or affecting the body as a whole. Reproductive issues included, but were not limited to, signs/ symptoms or concerns affecting the reproductive system including fertility, menopausal symptoms and menstrual disturbances

A) ***European Organisation for the Research and Treatment of Cancer (EORTC) QLQ-OV28)***: The EORTC Quality of Life Group’s core questionnaire, QLQ-C30, includes 30 questions relevant across all cancer sites, stage and treatments. It assesses five aspects of functioning (social, physical, role, cognitive and emotional functioning), eight symptoms (fatigue, nausea/vomiting, insomnia, pain, diarrhea, constipation, anorexia and dyspnoea) and includes questions relating to finances and overall HRQL [20]. It is complemented by the QLQ-OV28, a 28-item ovarian cancer-specific module assessing abdominal symptoms, peripheral neuropathy, chemotherapy-related side effects, hormonal symptoms, body image, sexual functioning and patients’ attitude to treatment [21]. Of these 28 questions, nineteen are symptoms and nine assess other aspects of HRQL.
B) ***Functional Assessment of Cancer Therapy-Ovarian Questionnaires (FACT-O)***: The FACT-O is the ovarian cancer-specific questionnaires within the Functional Assessment of Chronic Illness Therapy (FACIT) collection of HRQL instruments. It contains 39 items. The first 27 items assess physical, social, emotional and functional wellbeing. The remaining 12 items are ovarian cancer-specific, including seven symptoms and five other aspects of wellbeing [22].
C) ***National Comprehensive Cancer Network – FACT Ovarian Symptom Index 18 (NFOSI-18)***: After FACT-O was published, the National Comprehensive Cancer Network (NCCN) released an 18-item advanced ovarian cancer symptom index (NCCN-FACT Ovarian Symptom Index-18, otherwise known as NFOSI-18) [23]. This 18 item questionnaire was adapted from the FACT-O questionnaire and was developed to prioritise the symptoms considered most important by clinical experts and women with advanced ovarian cancer [23]. As such, NFOSI-18 was considered to address ovarian-cancer related HROL issues more specifically than FACT-0 [22].
D) ***The Quality of Life Instrument- Ovarian Cancer Patient Version (QOL-OVCA)***: The Quality of Life Instrument-Ovarian Cancer Patient Version, also known as the City of Hope Quality of Life Ovarian Cancer Tool (QOL-OVCA), is a 45-item ordinal inventory designed to measure HRQL in women with ovarian cancer [24]. The tool assesses four domains: physical, social, psychological and spiritual functioning [24].
E) ***The Measure of Ovarian Cancer Symptoms and Treatment (MOST)***: The Gynaecologic Cancer InterGroup (GCIG) established a working group to develop the most recent fit-for-purpose ovarian cancer-specific PROM tool – the ‘Measure of Ovarian Cancer Symptoms and Treatment’ (MOST) tool. The MOST tool was primarily designed to measure the benefit of palliative chemotherapy in patients with recurrent ovarian cancer in a clinical trial setting [25]. The first version of the MOST contains 35 items: 15 items assess symptoms of ovarian cancer and chemotherapy side-effects, two items assess anxiety and depression, three items assess physical, emotional and overall wellbeing, and the remaining 15 assess treatment-related concerns [25].

This questionnaire was intended to be flexible and modifiable, with inclusion and exclusion of specific items depending on clinical indication and target population [26]. The second version, MOSTv2 and more recent MOST-S-26, has been demonstrated to be fit-for-purpose for use in clinical trials of palliative chemotherapy as well as for patient follow-up [27]. It has 24 items and 5 multi-item scales: MOST-Well-being, MOST-Abdo (measuring abdominal symptoms), MOST-Psych (measuring psychological wellbeing), MOST-Chemo (measuring chemotherapy-related symptoms) and MOST-DorT (measuring disease or treatment-related symptoms) [26, 27]. An updated version of the MOST for use during follow-up/surveillance (MOST-S-26) has also recently been developed and validated [28].

### Psychometric properties of the above ovarian cancer-specific PROM tools

From the studies we identified analysing the psychometric properties of the five ovarian cancer-specific PROM tools, the metrics which were analysed included reliability, functionality, interpretability and validity [Table 2]. All tools met the minimum standard (i.e. adequate grading or above) as per the COSMIN guidelines for all four domains, with two exceptions. The FACT-O questionnaire only received a ‘doubtful’ grading with respect to interpretability and functionality, given many women found the questions about sexuality to be ‘intrusive,’ and there was no direct consumer input in selecting the items for inclusion in the tool. Furthermore, the QOL-OVCA tool received an ‘inadequate’ grading for interpretability and functionality given many consumers felt a number of items were poorly understood by respondents and consumers did not have direct input in selecting the items for inclusion. Notably, although all five tools were sufficiently validated for use in a clinical research setting, none of them have been validated for use in the clinic or within clinical quality registries [20, 21, 24, 25, 29].

Internationally, the two most widely used HRQL measures for ovarian cancer are the EORTC QLQ-OV28 and FACT-O questionnaires [30]. As these tools were two of the first ovarian cancer-specific PROM tools to be developed and have been extensively utilised in clinical trial settings, a stronger set of data exists examining their validity, reliability and responsiveness [30].

Given the MOSTv2 tool was developed after the existing systematic reviews comparing the ovarian-cancer specific PROM tools were published, we broadened our scope to include review articles [25, 31] in addition to systematic reviews [30, 32], so we could include data pertaining to all five ovarian cancer-specific PROM tools. Our literature review yielded two main systematic reviews and two review articles comparing ovarian cancer-specific PROM tools. A summary of the four main studies comparing these PROM tools and their findings are outlined in Table 1. Notably, all of these studies examine the ovarian cancer-specific PROM tools within the context of clinical trials (rather than a CQR).

Because the MOSTv2 tool was published in 2018, it has not yet been included in systematic reviews or other studies comparing the sensitivity and reliability of PROM tools for use in ovarian cancer [27]. However, the tool has only been validated for use in a very specific setting - trials of palliative chemotherapy in recurrent ovarian cancer [33]. The other tool which had not been extensively assessed in the reviews listed in Table 1 is the QOL- OVCA tool. Although it was developed in 1995, a paucity of evidence exists regarding the reliability and validity of QOL-OVCA. It has been less extensively tested for reliability and validity than its counterparts, the EORTC QLQ-OV28 and FACIT questionnaires [24, 34]. Further discussion on the interpretability, functionality, reliability and validity can be found in Table 2.

### Expert steering committee consensus

Compared to clinical trials, choosing a PROM tool for a CQR represents a unique challenge in needing to provide a set of questions relevant to all women with ovarian cancer, regardless of their treatment or stage of disease. Given all five tools have been designed and validated for use predominantly within a clinical trial setting, the decision of which tool to incorporate into NGOR was therefore based not on their ISOQOL or COSMIN ratings alone. Rather, an expert steering committee was convened to decide which tool would be most easily adaptable and functional for use within a CQR.

The committee identified a number of key purposes of collecting PROM data within the NGOR. The primary purpose was to enable cross-service comparison, benchmarking and assess variation among centres, predominantly at the ‘meso’ level to drive quality improvements of care at the organisational level. In the public health sphere, multilevel models that integrate the associations between distant and proximal outcomes of health can be defined at the micro level (i.e. the patient’s psychosocial and behavioural factors), the macro level (i.e. income distribution and welfare) and most importantly in our case, the meso level (i.e. institutional / organisational) [35]. To enable this, the data collected should be able to be benchmarked so stakeholders can assess if health services are meeting best practice standard of care. The committee also identified a key purpose of incorporating a PROMs tool within the CQR as being able to include the patient voice in the healthcare system, capturing the patients’ perspective of their wellbeing and utilising this data to determine whether these interventions actually make a difference to HRQL.

During the initial meetings, consensus was not reached on whether or not high level ‘quality of life’ data would be sufficient for this purpose or whether recording specific symptoms (e.g. post-operative pain) would also be relevant and valuable. Overall, the committee agreed that there should be a focus on these higher level QOL questions to be able to identify longer-term or important symptoms/side effects. Further, if the data was collected at the point of care, it could potentially be used to inform patient management on a micro level. This feedback to patients and treating clinicians would provide additional incentives for sustained PROM data collection; However, whether the data could be feasibly collected and used to inform real-time feedback was not clear at this stage of the development of the NGOR.

The steering committee identified the important selection criteria for the tool as being: short in length, easily comprehensible, acceptable and appropriate for women with ovarian cancer, provide coverage of the key HRQL impacts arising from diagnosis and treatments and available in a range of languages. The committee also agreed that if no single PROM tool provided the content required, either due to missing relevant content or containing irrelevant content, then if the PROM developer allowed items to be added, and irrelevant items to be removed, a bespoke tool could be created. Further the committee recommended that the tool should be applicable to a broad range of patients including marginalised, illiterate, non-English speaking patients, and those without computer access.

The five identified ovarian cancer-specific tools were each reviewed and discussed by the committee in great detail. The EORTC QLQ C30/OV28 provided the best fit to the agreed selection criteria. Together, they are arguably: among the most validated and well accepted tools by patients with ovarian cancer; widely used PROM in ovarian cancer care; and currently available in 55 languages with additional translations in progress [36]. Further, the EORTC QOL Group now supports user-created item lists selected from its item library [37] allowing a bespoke tool to be created. The QLQ-C30 and OV28 are designed to be used together and the combination of tools covers a broader range of highly important symptoms including gastrointestinal symptoms, depression, finances and sleep. Measuring PROMs in a CQR represents a unique challenge, whereby a tool needs to contain questions relevant to women across a variety of stages of their illness. For example, the tool needs to be relevant to women with metastatic disease receiving systemic treatment as well as women who have had localised resections or have been in remission for many years. The committee felt that including both a generic oncological tool (QLQ-C30) and a disease specific tool (OV28) would provide a balance of both depth and breadth of questions appropriate to women across all stages of their disease.

## Discussion

A search of the literature was conducted, and five ovarian-cancer specific PROM tools were identified; the EORTC QLQ-OV28, FACT-O, NSFOSI-18, QOL-OVCA and MOST. The reliability, validity, interpretability and responsiveness of the tools were graded according to the COSMIN guidelines. However, given that these metrics were analysed with the intention of the PROM tool being used with a clinical trial setting rather than within a CQR, the decision regarding which tool would be most adaptability for use within a CQR was made on the basis of expert steering committee consensus.

The expert steering committee identified the primary purpose of implementing a PROM tool into the NGOR as to enable cross-service comparison and benchmarking of care across centres and services through aggregated data at a meso level to drive organisational quality improvements. The committee also recommended that the chosen tool be short, easily comprehensible, acceptable, written in an appropriate form for women, be translatable into multiple different languages and assess both key symptoms/side-effects and higher-level data on QOL. The committee concluded that there is currently no single ‘best’ ovarian cancer-specific PROM tool, particularly for use in a CQR such as NGOR, however, that the EORTC tool would be most fit-for-purpose for incorporation into NGOR.

We are aware of the limitations of this review. Our review was limited to studies published in English, which may have impacted our understanding of which tools are more appropriate for culturally diverse populations. Tools that were developed more recently had fewer studies assessing their use and psychometric properties, thus their robustness and appropriateness for CQR inclusion is less certain. Importantly, none of the tools have been specifically validated for use within a CQR; though the role of our expert steering committee was to deduce this, a pilot study is needed to confirm whether a tool is appropriate for a CQR. In terms of the expert steering committee, though consumers, academic experts, and healthcare professionals were represented, we lacked representation from nurses and allied health professionals, who play key roles in the delivery of care for patients with ovarian cancer. There is a possibility that vital information from their perspective of care provision has been missed, however given the focus is on patient experiences and outcomes, we believe the consumer voice in our steering committee would capture the relevant content.

After reviewing each of the five ovarian cancer-specific PROM tools, the committee unanimously agreed that the EORTC tool was the most appropriate and fit-for-purpose tool for incorporation into the NGOR. Available in the largest variety of languages with translations being performed according to international best practice, the tool also met all the identified selection criteria and in particular, covered the greatest number of high priority quality indicators. The EORTC tool also provides the option of selecting individual items from either the EORTC QLQ C30 or QLQ-OV28 according to the requirements of the task.

The novel concept of using an expert-led steering committee to improve management and quality of care is fast becoming an increasingly used and valued approached, particularly within the field of oncology [38]. Our committee included an array of experts from a multitude of relevant fields, and critically, the two consumer representatives played a pivotal role in the committee.

Large-scale implementation of PROMs, such as on a national registry level, can be hampered by collection of poor-quality data, poor response rates and most notably, lack of consumer involvement in the developmental process [39]. The concept of incorporating patient involvement in the development of research is becoming increasingly recognised as a way to enhance the acceptability, quality and relevance of research – particularly when consumers are involved in all aspects of the research cycle from the development of the research questions to the dissemination of the findings [40].

Using consumers to guide research questions is exceptionally important when considering the development of a *patient-*reported outcome measure. Many agree that a validated PROM tool should involve patients in their development, but this involvement can sometimes be cursory and superficial [41]. This often involves patients sharing their experiences in a focus-group type setting, but them having little influence over the research aims, methodology, implementation and analysis [41]. When consumer involvement is more sustained and meaningful, particularly in the field of PROM development, this has been demonstrated to improve the acceptability, quality and relevance of the research [40].

As CQRs are increasingly being used to monitor and improve the quality of healthcare delivery and identify variations in clinical care [42], guidelines are increasingly recommending PROMs inclusions within CQRs [43]. In Australia, the Victorian Orthopaedic Trauma Outcome Registry and the Prostate Cancer Outcome Registry (PCOR) both collect PROMs and the Australian and New Zealand Thyroid Cancer Registry and Australasian Pelvic Floor Procedure Registry are considering incorporating them into use [43]. PCOR favoured the use of a prostate-cancer specific tool, the 26-item Expanded Prostate Cancer Index Composite survey (EPIC-26) [44]. However, there remains a paucity of available literature providing guidelines for the selection of PROM tools for use within a CQR. Further, implementation of PROMs into CQRs can be challenging, costly and time consuming [43]. An Australian research group has recently begun to develop preliminary guidelines on how to select and incorporate PROM tools within CQRs, including the recommendation to include both a generic and disease-specific instrument [43]. Although implementation requires clinical and operational resources and sufficient funding, the addition of PROM tools in a CQR can extend the scope and utility of the registry and improve shared decision making and treatment outcomes for patients [44].

Our steering committee was specifically designed to facilitate consumer input on all levels of research design, from identification of appropriate symptoms and metrics to guidance on logistics, feasibility, appropriateness and administration. This strong collaboration will be maintained throughout each phase of research development.

## Conclusions

The objective of this study was to identify ovarian-cancer specific PROM tools, perform a literature review of comparisons and analyses of the psychometric properties of the PROM tools and use an expert steering committee to determine which tool would be most fit-for-purpose for incorporation into NGOR.

Although the EORTC QLQ-C30/OV28 tool is a robust indicator of patient outcome measures, it is critical to determine if further content refinement is needed prior to the inclusion of these measures into the NGOR. Future work will involve creating this bespoke assessment tool. Phase One of this work will include assessing which items from the EORTC QLQ-C30/OV28 tool are deemed important to include in the CQR by women diagnosed with ovarian cancer. Consumers can indicate which items in the questionnaire are relevant and important to their experiences and concurrently, which questionnaire items are perhaps superfluous. Phase Two will confirm that the bespoke instrument constructed from phase 1 results captures all the important outcomes and is acceptable in terms of content and length as well as frequency of administration of these questionnaires and preferred methods of completion. Addressing these needs may ensure that the PROM data captured is relevant and meaningful to women in a way that allows for a more patient-centred assessment of treatment efficacy and disease trajectory.

By incorporating a PROM tool, the NGOR will hopefully be able to more effectively evaluate treatments, monitor symptoms that are likely to impact on QOL and inform clinical decision making in the management of ovarian cancer [41] although how this information will be used with benchmarked clinical data requires further study. The mode, method, timing and logistics of the administration of the tool will also be determined after the creation of the bespoke tool using consumer input as a driving influence.

In collaboration with Ovarian Cancer Australia (OCA), we are in the process of conducting qualitative research with consumers to identify the sentinel quality of life issues for women with ovarian cancer and select items from the EORTC tools for implementation into a CQR. This approach will hopefully enable the measurement of the quality of care and drive optimal outcomes in Australia’s multicultural population of women.

## Data Availability

Excel spreadsheet of literature review and minutes from NGOR PROM steering committee available upon editor’s request.

## Abbreviations

CONSORT: Consolidated Standard of Reported Clinical Trials
COSMIN: Consensus–Based Standards for the selection of health Measurement Instruments
CQR: Clinical Quality Registries
EORTC QLQ: OV28–European Organisation for the Research and Treatment of Cancer ovarian cancer module
ESMO: European Society for Medical Oncology
FACT: O–Functional Assessment of Cancer Therapy Ovarian questionnaires
FDA: The U.S Food and Drug Administration
HRQL: Health related quality of life
ICHOM: International Consortium for Health Outcomes Measurement
ISOQOL: International Society for Quality of Life Research
MOST: Measure of Ovarian Cancer Symptoms and Treatment
MOSTv2: Measure of Ovarian Cancer Symptoms and Treatment version 2
NCCN: National Comprehensive Cancer Network
NFOSI: 18–National Comprehensive Cancer Network–FACT Ovarian Symptom Index 18
NGOR: National Gynae–Oncology Registry
NHIPPC: National Health Information and Performance Principal Committee
OCA: Ovarian Cancer Australia
PCOR: Prostate Cancer Outcome Registry
PROM: Patient Reported Outcome Measure
QOL: Quality of life
QOL: OVCA–Quality of Life Instrument–Ovarian Cancer Patient Version
UGICR: Upper Gastrointestinal Cancer Registry
